# Nutrients Leaching in Response to Long-Term Fertigation and Broadcast Nitrogen in Blueberry Production

**DOI:** 10.3390/plants9111530

**Published:** 2020-11-10

**Authors:** Aimé J. Messiga, Kathryn Dyck, Kiera Ronda, Kolden van Baar, Dennis Haak, Shaobing Yu, Martine Dorais

**Affiliations:** 1 Agassiz Research and Development Centre, Agriculture and Agri-Food Canada, 6947 Highway 7, P.O. Box 1000, Agassiz, BC V0M 1A0, Canada; Kathryn.Dyck@mytwu.ca (K.D.); kiera.ronda11@hotmail.ca (K.R.); koldenv@uvic.ca (K.v.B.); adhaak@shaw.ca (D.H.); shaobing.yu@canada.ca (S.Y.); 2 Centre de Recherche et d’Innovation sur les Végétaux (CRIV), Département de Phytologie, Faculté des Sciences de l’Agriculture et de l’Alimentation, Université Laval, Québec (Québec), G1V 0A6, Canada; martine.dorais@fsaa.ulaval.ca

**Keywords:** ammonium, British Columbia, leachates, nitrates, sawdust mulch, sulfate

## Abstract

Nutrient leaching losses from horticultural production threaten the quality of groundwater and freshwater systems worldwide. The objectives of this study were to (a) assess the effects of annual applications of ammonium sulfate fertilizer through fertigation (FERT) and broadcast (BROAD) on nutrient leaching losses and (b) determine the links among chemical property changes in leachates and soil with berry yields after 9 and 11 years of blueberry production. The long-term blueberry site was established in 2008 using seven combinations of treatments including an unfertilized control (CONT) and three N fertilizer rates (100%, 150%, 200% of recommended rates) using BROAD and FERT methods. Nutrients concentrations (NO_3_^−^-N, NH_4_^+^-N and SO_4_^2−^-S) and chemical properties (pH and electrical conductivity (EC)) of leachate, sawdust and soil and berries were assessed. All FERT methods resulted in concentrations of NO_3_^−^-N in the leachates > 100 mg L^−1^ with a maximum of 200 mg L^−1^ for FERT-200 during the growing season due to the easy transport of dissolved nutrients with the irrigation water. All BROAD methods resulted into concentrations of NO_3_^−^-N in the leachates >10 mg L^−1^ with a maximum of 35 mg L^−1^ for BROAD-200 between April and July, as well as between November and April, indicating two periods of NO_3_^−^-N leaching losses. The pattern observed with BROAD indicates that irrigation water in the summer and heavy rainfall in the winter contribute to NO_3_^−^-N leaching losses. Concentrations of NH_4_^+^-N in the leachates >1 mg L^−1^ were measured under FERT with a peak at 64.78 mg L^−1^ for FERT-200, during the period April to August, due to NH_4_^+^’s ability to quickly move through the sawdust layer with irrigation water. Principal component analysis linked berry yield decrease with ammonium sulfate applications above recommended rates (FERT and BROAD) and with changes in soil pH and EC. Our results demonstrated that excess fertilizer applications above recommended rates using FERT and BROAD can threaten the sustainability of blueberry production by enhancing nutrient leaching losses and reducing berry yield.

## 1. Introduction

Nutrient leaching losses from horticultural production threaten the quality of groundwater and freshwater systems worldwide [1]. One of the main drivers of nutrient leaching is the excessive use and improper management of fertilizers that provide nutrients in amounts larger than the crop needs. Horticultural production systems are high value-added crops and farmers apply excess fertilizers because the probability of economic return is high [2]. A study conducted in Chinese farmland showed that nitrogen (N) application rates in orchards were often higher than (1) the recommended N application rate and (2) the economic optimum N application rates [3]. In a 3-year study involving 29 raspberry trials in farmer fields in the Lower Fraser Valley of British Columbia (BC), Canada, high soil nitrate (NO_3_^−^-N) contents were frequently measured at the end of the growing season even at the recommended N rate, which indicates excess N fertility [4]. Excessive fertilizer applications can lead to high concentrations of nutrients in the soil and the nutrients not taken up by plants are prone to leaching.

Nutrient leaching losses can affect groundwater and freshwater systems in various ways. High NO_3_^−^-N loads can trigger eutrophication of surface waters and result in algal blooms and loss of fish [5]. Methemoglobinemia in infants and some forms of stomach cancer in adults have been linked to high concentrations of NO_3_^−^-N in drinking water [6]. These concerns have led to policies requiring farmers to adopt improved N management practices in areas sensitive to NO_3_^−^-N leaching and the establishment of critical NO_3_^−^-N concentration limits for drinking water [7,8]. Studies have also shown that ammonium (NH_4_^+^-N) may leach in sandy soils [9]. High concentrations of total ammonia (>1 mg L^−1^) in freshwater environments can be toxic to aquatic and semiaquatic plant species [10]. Fish species, such as *Oncorhynchus mykiss* (rainbow trout), were found to be negatively affected at ammonia concentrations as low as 0.04 mg L^−1^ [11]. Sulfate (SO_4_^2−^-S) leaching in horticultural production generally occurs as a result of excessive use of ammonium sulfate fertilizers [12]. It accumulates in the soil as a salt, which increases electrical conductivity (EC) and acidity [13]. Concentrations of SO_4_^2−^-S in the soils also depend on the dynamics of soil organic matter, as well as precipitation and temperature patterns during winter and spring [14]. Results from lysimeters and river catchment studies showed that SO_4_^2−^-S leaching ranges from 1 to 60 kg ha^−1^ yr^−1^ [15], but values as high as 100 kg ha^−1^ yr^−1^ have also been reported [16].

Blueberries (*Vaccinum corymbosum*) grow best in acidic soils with optimal pH between 4.0 and 5.5 [17]. They take up N preferentially as NH_4_^+^ compared with NO_3_^−^, and therefore blueberry farmers in most of BC use ammonium sulfate as their main source of N [13]. As for most horticultural crops, blueberries are high-value crops and surveys have shown that blueberry farmers in BC generally apply ammonium sulfate fertilizers at rates greater than recommended [13,18]. Another explanation for the excess N input in these horticultural production systems is the use of sawdust mulch, a carbon-rich material, on the surface of raised beds to improve weed control and limit evaporation of soil moisture and protect the roots from extreme temperatures because blueberry plants have a shallow root system [19]. Messiga et al. [13] found that ammonium sulfate fertilizer applications above recommended N rates increased NO_3_^−^-N concentrations in the 0–30 cm soil layer by 2.6. to 3.6 times under fertigation (FERT), while NH_4_^+^-N concentrations increased in the sawdust mulch layer by 1.9 times. In general, when N input is greater than the optimal rate, yield may not increase further, but nutrient leaching may increase linearly or exponentially [20].

The shallow and confined root system of blueberry plants restricts their capacity to take up water from the soil and therefore irrigation is a key factor in the growth and production [19]. Drip irrigation is common in blueberry plantings and it is efficient because it reduces the amount of water required compared to overhead sprinklers [19]. However, irrigation can contribute to nutrient leaching, causing NO_3_^−^-N, NH_4_^+^-N and SO_4_^2−^-S derived from ammonium sulfate fertilizer to drip downward through the soil profile and pose a great threat to the quality of groundwater [21]. Drip irrigation is also suitable for fertigation, but studies have shown that nutrient leaching can be increased with fertigation because dissolved nutrients move easily through the soil with water [13]. Ehret et al. [22] showed that NH_4_^+^ leaching increases greatly in blueberry planting with fertigation, especially at higher rates, because of the ability to move quickly through the layer of sawdust mulch.

In a recent study, we showed that the use of ammonium sulfate fertilizer with fertigation above recommended rates can cause berry yield and soil pH to decrease, as well as increases in soil EC [13]. How nutrient leaching is influenced by N fertilizer rates and application methods and whether decreased berry yield trends with N applications above recommended N rates observed under fertigation will appear under broadcast (BROAD) with continuous ammonium sulfate fertilizer applications later in the production cycle is still unknown from this long-term blueberry planting. Adu-Gyamfy et al. [23] used ceramic cup lysimeters to assess nutrient leaching from corn production systems in northern Ghana. Similarly, Zvomuya et al. [24] studied nitrate leaching in potato fields in Minnesota. Ceramic cup lysimeters provide a way to assess nutrient leaching and these were considered in the present study. The objectives of this study were to (a) assess the effects of annual applications of ammonium sulfate fertilizer through fertigation and broadcast applications on nutrient leaching losses, including NO_3_^−^-N, NH_4_^+^-N and SO_4_^2−^-S and (b) determine the links among chemical property changes in soil and leachates with berry yields.

## 2. Materials and Methods

### 2.1. Site Description

The long-term blueberry site was established in 2008 at Agassiz Research and Development Centre (Agriculture and Agri-Food Canada) (49°14′ N, 121°45′ W). Several papers have already been published from this site on a wide range of topics [13,18,22]. The local climate of the Fraser Valley is characterized as moderate oceanic with relatively cool, dry summers and warm, rainy winters; the average annual rainfall is 1689 mm, where 261.9 mm falls between May and July. The local climate occupies a narrow annual temperature range between 3.2 °C in December and 18.7 °C in August. The topography of the area is relatively flat with an average elevation of 7.6 m above sea level. The soil at the site is a silt loam soil of the Monroe series and is moderately well drained (Typic Dystroxerepts under the U.S. Soil Taxonomy [25]. Until 2006, the field received compost derived from lawn clippings and poultry and greenhouse vegetable waste. The chemical characteristics of the topsoil when the experiment was established were: 5.27% organic matter and 29 kg ha^−1^ soil mineral N [13,22]. Field preparation and planting occurred in the spring and fall, respectively, in 2008 [13].

### 2.2. Experimental Design and Treatments

Seven combinations of treatments, including an unfertilized control (CONT), three N fertilizer rates (100%, 150%, 200% of recommended rates in the British Columbia Berry Production Guide [26]) and two application methods (broadcast (BROAD) and fertigation (FERT)), were compared. The seven combinations of treatments (CONT, BROAD-100, FERT-100, BROAD-150, FERT-150, BROAD-200 and FERT-200) were applied to a randomized complete block design with four replicates for a total of 28 experimental units. The experimental units consisted of seven plants, including five measurement plants and two guard plants at the edges of the plots. The N fertilizer used was ammonium sulfate (21-0-0). Nitrogen application via BROAD was applied at the surface of the sawdust mulch surrounding the base of the blueberry plants and occurred over a span of eight weeks, beginning in late April or early May. A total of three split applications were applied in which one BROAD application was equivalent to one third of the total annual N. Nitrogen applications via FERT began at bud break and were repeated every week (ending in late August or early September). Fifteen equal applications were used for FERT so that each application consisted of 1/15 of the total annual N. Total N applications every year were 144 kg N ha^−1^ for 100% (recommended rate, [26], 215 kg N ha^−1^ for 150% and 287 kg N ha^−1^ for 200% [18]. Each treatment (FERT, BROAD and CONT) received an equal amount of water; however, FERT application and irrigation did not occur simultaneously, but were completed within 7 h periods of each other.

Fertigation and irrigation were applied through two lines of drip tape (DLT Heavywall Dripperline, Netafim, Fresno, CA, USA) equipped with emitters. Granular matrix sensors (Watermark Model 900 M, Irrometer Co., Riverside, CA, USA) were used to monitor soil moisture tension and EC-5 sensors (Decagon Devices Inc., Pullman, WA, USA) for soil water content. Plants are pruned according to industry standards every year [26]. Following the 2016 harvesting season, the plants were heavily pruned to rejuvenate the plantings. A honeybee hive was installed to encourage pollination and bird netting placed around the bushes to prevent berry loss. Pesticides were applied for weed, fungus (botrytis blossoms) and insect (Bruce spanworm (*Operophtera bruceata*) and European leafroller (*Archips rosanus*)) control [13,22].

### 2.3. Leachate Samples

Leachate samples were collected from all experimental plots using ceramic cup (0.1 MPa air entry pressure) suction lysimeters (Soilmoisture Equipment Corp., Santa Barbara, CA, USA) during the periods of 30 March 2016–20 February 2017 and 22 March 2017–17 April 2018. The suction lysimeters were assembled in the laboratory by fixing the round bottom, porous ceramic cup to one end of a 60 cm polyvinyl chloride (PVC) tube [27]. One suction lysimeter was installed in the middle of each plot at a 76 cm depth below the soil surface, to capture residual nutrients leached from the rooting zone, as described by Zvomuya et al. [24]. Briefly, at the point of the installation, a hole was dug vertically at a 76 cm depth using an auger. Two hundred and fifty milliliters of silica flour were poured into the bottom of the hole to improve the hydraulic contact between the porous ceramic cup and the surrounding soil. The suction lysimeter was then inserted into the hole and the soil augured out was repacked and the lysimeter was sealed at the soil surface with bentonite to reduce water flow along the shaft.

To collect leachate samples, a suction pressure of 40 kPa was applied with a hand pump and the lysimeters were sealed for 24 h. Previous studies showed that a 40 kPa vacuum was sufficient to create suction in the cup above that of the surrounding soil until the sample was extracted [24]. After 24 h, leachate samples were collected from the lysimeters using the hand pump and 100 mL of the leachate was kept frozen until analysis.

### 2.4. Sawdust Mulch and Soil Sampling

Sawdust and soil samples were collected in fall 2018. The sawdust layer, approximately 10 cm thick, was collected by hand. Four soil cores (2 cm diameter) were then collected from between the measurement plants, along the dripline, at depths of 0–30 cm and 30–60 cm. The field moist sawdust and soil samples were composited on-site, air-dried, sieved (2 mm) and stored at room temperature until analysis.

### 2.5. Berry Yield

Yield data presented in this study represent three consecutive years of harvest starting in 2016 and ending in 2018. Yield data from previous years are presented elsewhere [13,22]. Berries were harvested twice a year from the five measurement plants of each experimental plot and weighed for fresh berry yield [13]. Berries were hand-picked in late June for the first harvest and mid-July for the second harvest. During the second harvest, unripe fruits were hand-picked from the bushes to avoid a third picking, due to limited resources. The sum of berry yields in the first and second harvests, including unripe fruits, represents total berry yield.

### 2.6. Chemical Analysis

The concentrations of NO_3_^−^-N and NH_4_^+^-N in the leachate samples were analyzed by the colorimetric method using a flow injection analyzer (Tecator FIAStar 2010) as described by Maynard et al. [28]. The concentration of SO_4_^−^-S in the leachate samples was analyzed by the turbidimetric method [29]. The pH and EC of the leachate samples were measured directly in a 20 mL volume using a pH/EC meter (YSI MultiLab IDS 4010-3W). Soil mineral N (NO_3_^−^-N and NH_4_^+^-N) was analyzed by extracting 5 g subsamples of air-dried soil and 5 g of sawdust using 2 M KCl in a 1:10 (*w:v*) ratio. The colorimetric analysis of the soil samples for NO_3_^−^-N and NH_4_^+^-N was performed using a flow injection analyzer [28]. A 1:1 (*w*/*v*) soil:solution ratio was used to measure the soil pH and EC of the air-dried samples in distilled water.

### 2.7. Statistical Analysis

The SAS univariate procedure was used to test for normality and the analysis of variance (ANOVA) was performed with SAS Proc Mixed, version 9.3 [30] for all data. For all parameters, replicates and years were considered random and repeated effects, respectively. Treatments and two-way interactions (treatments for berry yield; treatments and treatments × sampling dates for leachate properties; treatments and treatments × depth for mineral N and soil properties) were considered fixed effects where appropriate. Differences among least square means (LSMEANS) for all treatment pairs were tested at a significance level of *p* = 0.05. Where appropriate, LSMEANS were compared with a combination of orthogonal and polynomial contrasts: CONT versus ALL; FERT versus BROAD; linear and quadratic effects for N rates. Principal component analysis (PCA) was carried out using SAS Proc Princomp to establish the relationships between yields and chemical properties of soil and leachate.

## 3. Results

### 3.1. Weather Conditions

**Figure 1:**
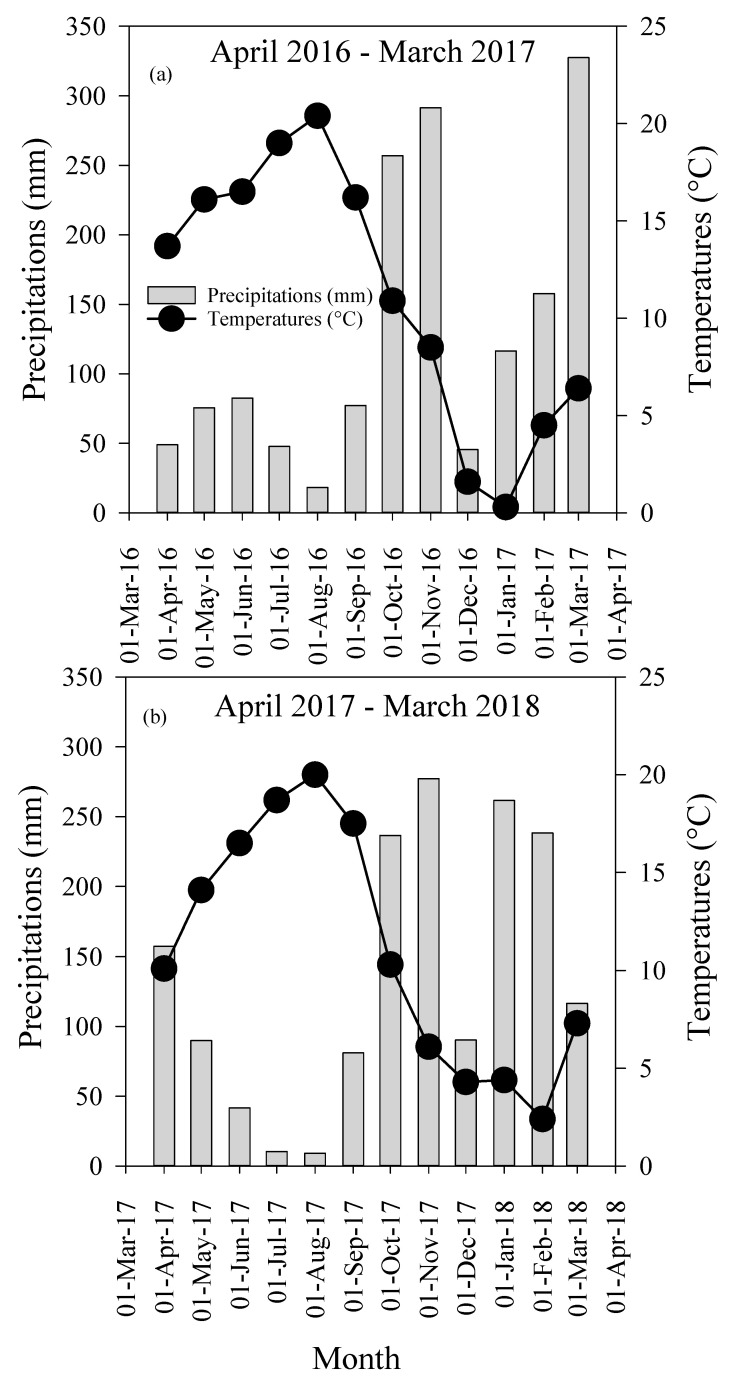
Average monthly precipitation and air temperatures during the periods (**a**) April 2016–March 2017 and (**b**) April 2017–March 2018.

Total precipitation was 1546 mm during the 2016/17 period (March 2016–February 2017) and 1610 mm during the 2017/18 period (March 2017–February 2018) (Figure 1). The growing season, from May to September, received 302 mm of rainfall during the 2016/17 period, but 232 mm during the 2017/18 period. More specifically, June with 83 mm, July with 48 mm and August with 18 mm during the 2016/18 period received 2.0, 5.0 and 2.0 times more rainfall compared with the same months during the 2017/18 period, while May and September received similar rainfall during the two periods. For the non-growing season, (October to April), the 2016/17 period received 25 mm less precipitation than the 2017/18 period. More specifically, December with 90 mm, January with 261 mm and February with 238 mm during the second period received 2.0, 2.3 and 1.5 times more precipitation compared with the same months of the 2016/17 period. In contrast, April 2017 received 3.0 times more precipitation compared with April 2016, while March 2017 received 2.8 times more precipitation compared with March 2018. The mean growing season air temperatures were 17.64 °C and 17.36 °C during the 2016/17 and 2017/18 periods, respectively (Figure 1a,b). The mean non-growing season air temperatures were 5.37 °C and 5.80 °C during the 2016/17 and the 2017/18 periods, respectively.

### 3.2. Concentrations of Nitrate, Ammonium and Sulfate in Leachate

**Figure 2:**
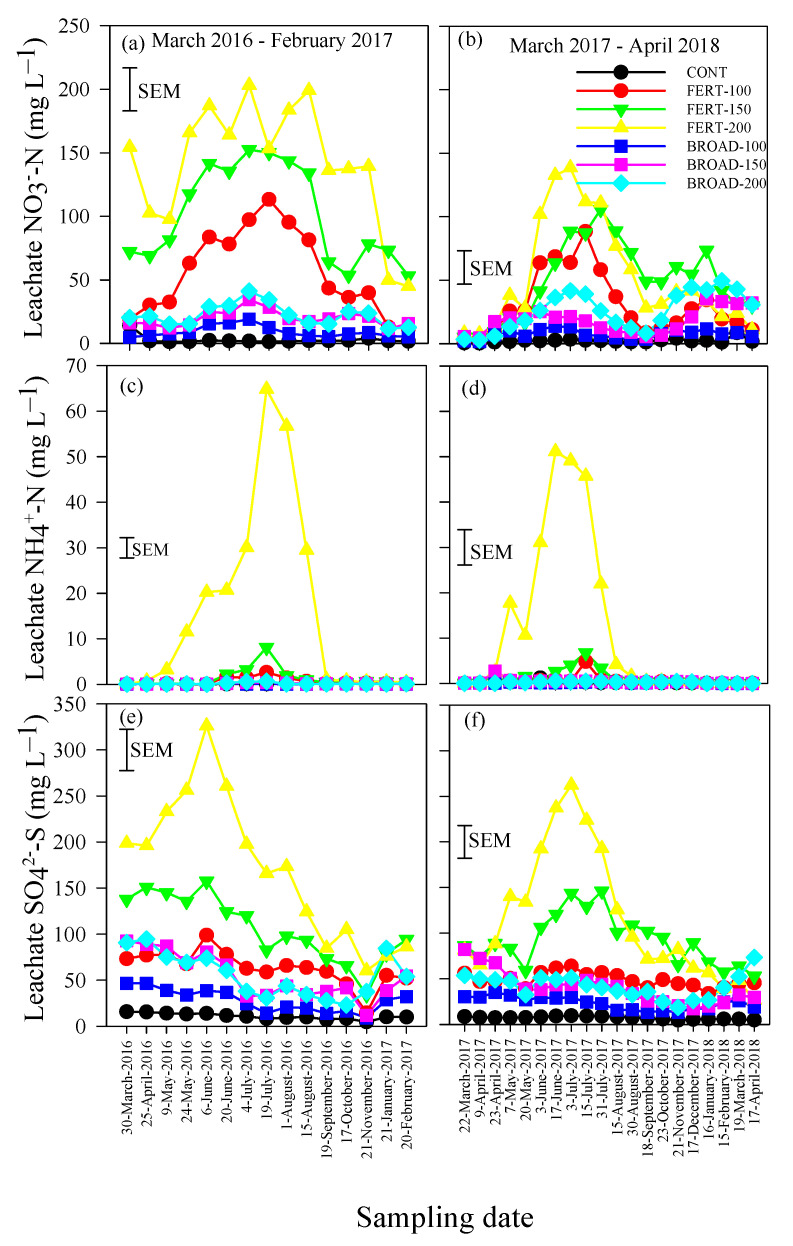
Concentrations of (**a**,**b**) nitrate nitrogen (NO_3_-N), (**c**,**d**) ammonium nitrogen (NH_4_-N) and (**e**,**f**) sulfate sulfur (SO_4_-S) in the leachate collected at 76 cm soil depth with annual applications of N through the fertigation (FERT-100, 100%; FERT-150, 150%; FERT-200, 200%) and broadcast (BROAD-100, 100%; BROAD-150, 150%; BROAD-200, 200%) methods to highbush blueberry (*Vaccinium corymbosum*) for two consecutive years (March 2016–February 2017 and March 2017–April 2018). Error bars represent the standard errors of the means (SEM) for comparing all values (n = 420 and df = 294).

The concentrations of NO_3_^−^-N in the leachate were influenced by N application methods and rates, but the extent varied with sampling periods (Table 1). In the 2016/17 period, concentrations of NO_3_^−^-N > 10 mg L^−1^, which are equivalent to the Canadian critical limit of NO_3_^−^-N in drinking water [7], were measured throughout sampling dates for FERT-based applications and BROAD-150 and BROAD-200 (Figure 2a). Concentrations of NO_3_^−^-N > 100 mg L^−1^ were measured between 30 March and 21 November for FERT-200 (102.75–203.14 mg L^−1^), 9 May to 19 September for FERT-150 (117.71–152.51 mg L^−1^) and 4 July to 1st August for FERT-100 (113.4 mg L^−1^) (Figure 2a). In the 2017/18 period, concentrations of NO_3_^−^-N > 10 mg L^−1^ were measured for FERT-based and BROAD-200 applications between 23 April 2017 and 17 April 2018 (Figure 2b). For BROAD-150, concentrations of NO_3_^−^-N > 10 mg L^−1^ were measured between 21 November and 17 April 2018 (11.57–35.33 mg L^−1^) and 7 May and 31 July 2017 (10.25–13.75 mg L^−1^), while this was observed between 21 November and 16 January 2018 (10.78–11.72 mg L^−1^) for BROAD-100 and between 23 April and 31 July 2017 (12.57–25.29 mg L^−1^) (Figure 2b). Concentrations of NO_3_^−^-N > 100 mg L^−1^ were measured between 3rd June and 31 July 2017 for FERT-200 (101.82–138.58 mg L^−1^) and 31 July 2017 for FERT-150 (105.79 mg L^−1^). CONT had a lower concentration of NO_3_^−^-N compared with all the other N application rates and BROAD-based application had lower NO_3_^−^-N levels compared with FERT-based applications (Table 1).

The concentration of NH_4_^+^-N in the leachate was also influenced by N application methods and rates, but the extent varied with sampling dates (Table 1; significant interactions). In the 2016/17 period, concentrations of NH_4_^+^-N > 1.00 mg L^−1^, which can be toxic to aquatic and semiaquatic plant species [10], were measured between 20 June and 1st August 2016 for FERT-100 with a peak at 2.61 mg L^−1^ and FERT-150 with a peak at 8.12 mg L^−1^, but between 9 May and 19 September 2016 for FERT-200 with a peak at 64.78 mg L^−1^ (Figure 2c). In the 2017/18 period, concentrations of NH_4_^+^-N > 1.00 mg L^−1^ were measured on 15 July 2017 for FERT-100 with a peak at 4.82 mg L^−1^, between 20 May and 31 July 2017 for FERT-150 with a peak at 6.77 mg L^−1^, but between 23 April and 30 August 2017 for FERT-200 with a peak at 51.11 mg L^−1^ (Figure 2d). The concentrations of NH_4_^+^-N remained <1.00 mg L^−1^ for the other N applications throughout the two sampling periods. CONT had a lower concentration of NH_4_^+^-N compared with all the other N application rates and BROAD-based applications had lower NH_4_^+^-N levels compared with FERT-based applications (Table 1).

The concentrations of SO_4_^−^-S in the leachate was influenced by N applications, but the extent varied with time in 2016/17 and 2017/18 (Table 1). In the 2016/17 period, the concentrations of SO_4_^−^-S varied between 4.70 mg L^−1^ for CONT on 21 November 2016 and 326 mg L^−1^ for FERT-200 on 6th June 2016 (Figure 2e). Concentrations > 100 mg L^−1^ were measured between 30 March and 4 July 2016 for FERT-150 (120.00–157.50 mg L^−1^) and between 30 March and 17 October 2016 for FERT-200 (104.90–326.00 mg L^−1^). In the 2017/18 period, the concentrations of SO_4_^−^-S varied between 4.60 mg L^−1^ for CONT on 21 November and 262.10 mg l^−1^ on 3 July 2017 (Figure 2f). Concentrations >100 mg L^−1^ were measured between 3^rd^ June and 18 September 2017 for FERT-150 (101.80–146.03 mg L^−1^) and between 7th May and 15 August 2017 for FERT-200 (125.70–262.10 mg L^−1^). CONT had a lower concentration of SO_4_^−^-S compared with all the other N application rates and BROAD-based applications had lower SO_4_^−^-S levels compared with FERT-based applications (Table 1).

### 3.3. Acidity and Electrical Conductivity of Leachate

**Figure 3:**
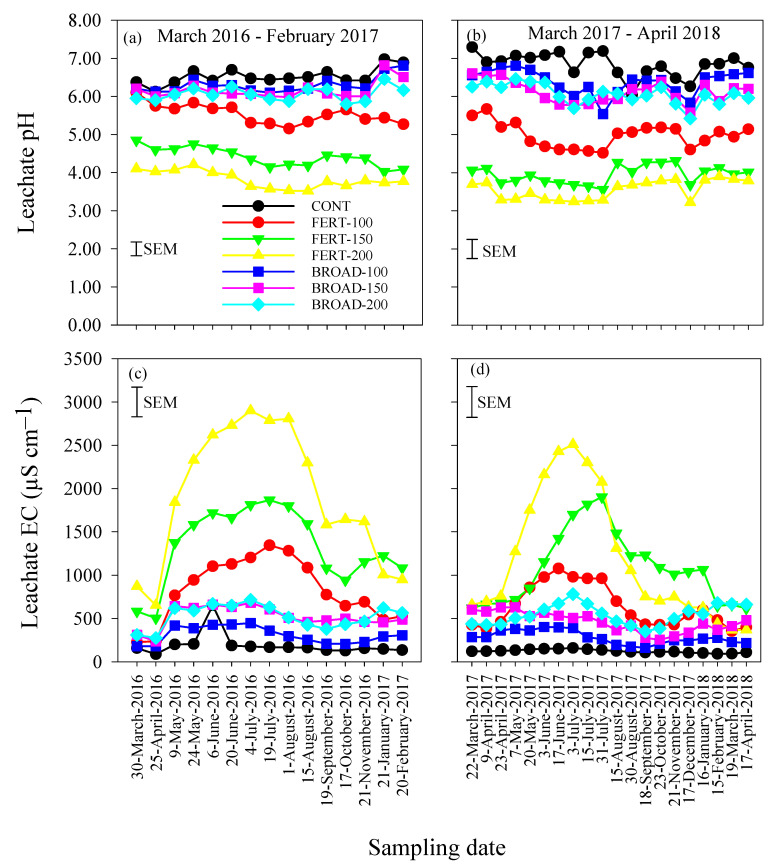
(**a**,**b**) Acidity (pH) and (**c**,**d**) electrical conductivity (EC) of leachate collected at 76 cm soil depth with annual applications of N through fertigation (FERT-100, 100%; FERT-150, 150%; FERT-200, 200%) and broadcast (BROAD-100, 100%; BROAD-150, 150%; BROAD-200, 200%) methods to highbush blueberry (*Vaccinium corymbosum*) for two consecutive years (March 2016–February 2017 and March 2017–April 2018). Error bars represent the standard errors of the means (SEM) for comparing all values (n = 420 and df = 294).

The pH of leachate was influenced by N application methods and rates, but the extent varied with the sampling periods (Table 1). During the 2016/17 period, the pH of leachate varied between 3.52 (FERT-200) on 15 August 2016 and 6.97 (CONT) on 21 January 2017 (Figure 3a). The pH of leachate ranged between 6.10 and 5.16 for FERT-100, 4.85 and 4.03 for FERT-150 and 4.21 and 3.52 for FERT-200 and 5.80 and 6.45 for BROAD-200 (Figure 3a). The pH values > 6.00 were measured under CONT, BROAD-100 and BROAD-150 (Figure 3a). During the 2017/18 period, the pH of leachate varied between 3.22 for FERT-200 on 17 December 2017 and 7.30 for CONT on 22 March 2017 (Figure 3b). The pH values of the leachate ranged between 5.67 and 4.52 for FERT-100, 3.56 and 4.32 for FERT-150 and 3.90 and 3.22 for FERT-200 (Figure 3b). The pH values were >6.05 under CONT, while pH values ranging between 5.42 and 6.81 were measured in leachate collected under BROAD-based treatments (Figure 3b). CONT had higher pH compared with all the other N application rates and BROAD-based applications had higher pH compared with FERT-based applications (Table 1).

The EC of leachate was influenced by N application methods and rates, but the extent varied with time in 2016/17 and 2017/18 (Table 1). In the 2016/17 period, EC values > 760 µS cm^−1^ were measured in the leachate collected from 9 May 2016 and 19 September 2016 under FERT-100 (767–1381 µS cm^−1^), and on 20 February 2017 under FERT-150 (943–1867 µS cm^−1^) and FERT-200 (872–2900 µS cm^−1^) (Figure 3c). In the 2017/18 period, EC values > 760 µS cm^−1^ [13] were measured in the leachate collected from 20 May and 31 July 2017 under FERT-100 (860–1078 µS cm^−1^) and 16 January 2018 under FERT-150 (850–1903 µS cm^−1^), and as early as 23 April and 30 August 2017 under FERT-200 (760–2510 µS cm^−1^) (Figure 3d). The EC of leachate collected under CONT and BROAD-based N applications remained lower than 760 µS cm^−1^ (Figure 3c,d). CONT had lower EC compared with all the other N application rates and BROAD-based applications had lower EC compared with FERT-based applications (Table 1).

### 3.4. Ammonium, Nitrate and Mineral N in the Soil

**Figure 4:**
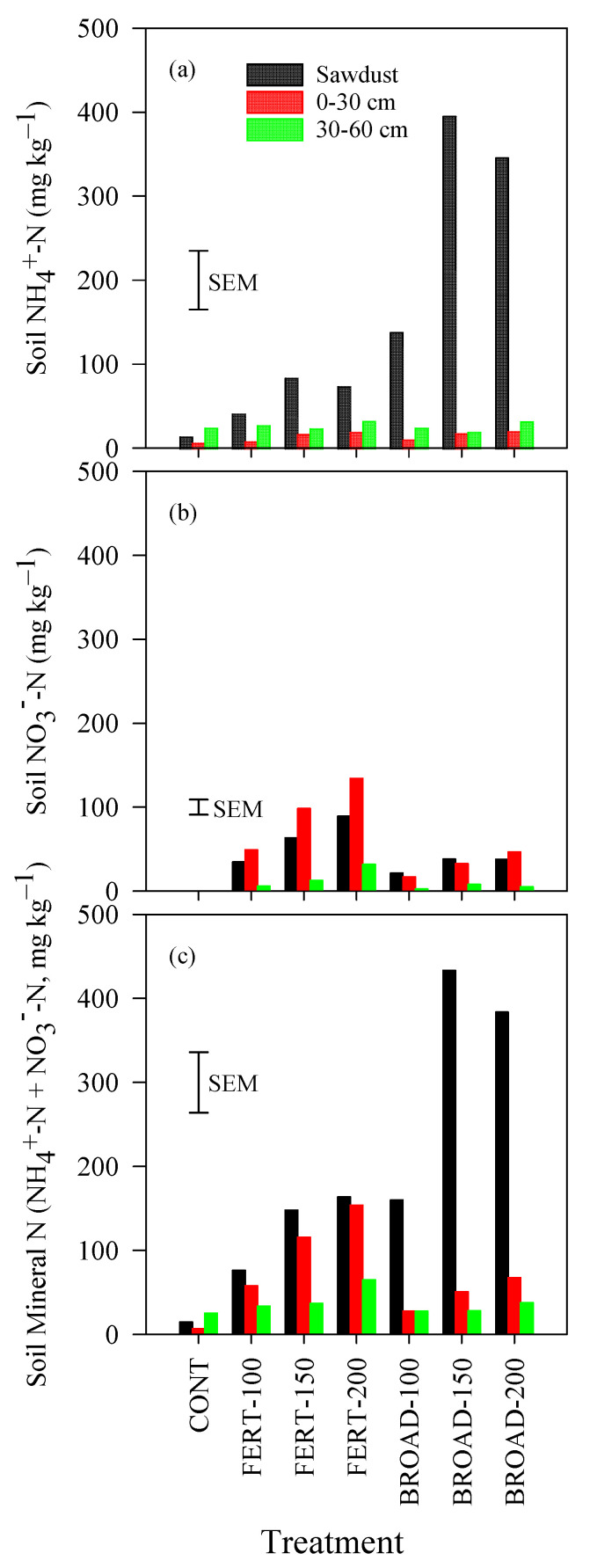
Concentrations of (**a**) Ammonium nitrogen (NH_4_^+^-N), (**b**) nitrate nitrogen (NO_3_^−^-N) and (**c**) mineral nitrogen (NH_4_^+^-N + NO_3_^−^-N) at 0–30 cm and 30–60 cm soil depth and in the sawdust mulch layer with annual applications of N through fertigation (FERT-100, 100%; FERT-150, 150%; FERT-200, 200%) and broadcast (BROAD-100, 100%; BROAD-150, 150%; BROAD-200, 200%) methods to highbush blueberry (*Vaccinium corymbosum*) in fall 2018. Error bars represent the standard errors of the means (SEM) for comparing all values (n = 28 and df = 21).

The concentration of NH_4_^+^-N was significantly influenced by N application methods and rates, but the extent varied with soil depth (*p* = 0.002) (Table 2). A quadratic effect of the N application rates was observed. In the sawdust layer, the concentration of NH_4_^+^-N varied from 14.62 mg kg^−1^ under CONT to 395.32 mg kg^−1^ under BROAD-150; in the 0–30 cm depth, the concentration of NH_4_^+^-N varied from 7.02 mg kg^−1^ under CONT to 21.07 mg kg^−1^ under BROAD-200; in the 30–60 cm soil depth, the concentration of NH_4_^+^-N varied from 25.05 mg kg^−1^ under CONT to 33.08 mg kg^−1^ under FERT-200 (Figure 4a).

The concentration of NO_3_^−^-N was significantly influenced by N application methods and rates, but the extent varied with soil depth (*p* = 0.020) (Table 2). A linear effect of the N application rates was observed. In the sawdust layer, the concentration of NO_3_^−^-N varied from 0.00 mg kg^−1^ under CONT to 89.43 mg kg^−1^ under FERT-200; in the 0–30 cm soil depth, the concentration of NO_3_^−^-N varied from 0.00 mg kg^−1^ under CONT to 134.16 mg kg^−1^ under FERT-200; in the 30–60 cm soil depth, the concentration of NO_3_^−^-N varied from 0.16 mg kg^−1^ under CONT to 31.80 mg kg^−1^ under FERT-200 (Figure 4a). Orthogonal contrast comparisons showed that the concentrations of NO_3_^−^-N were lower under CONT compared with all N application rates, while the concentrations of NO_3_^−^-N were higher under FERT-based compared with BROAD-based (32.50 mg kg^−1^ under sawdust, 31.95 mg kg^−1^ under 0–30 cm depth, and 5.15 mg kg^−1^ under 30–60 cm depth) treatments (Table 2).

The concentration of soil mineral N (NH_4_^+^-N + NO_3_^−^-N) was significantly influenced by N application methods and rates, but the extent varied with soil depth (*p* = 0.002). More specifically, there was a linear trend of soil mineral N with N application rates (Table 2). In the sawdust layer, the concentration of soil mineral N varied from 14.62 mg kg^−1^ (CONT) to 433.38 mg kg^−1^ (BROAD-150); in the 0–30 cm soil depth, the concentration of soil mineral N varied from 7.02 mg kg^−1^ (CONT) to 153.94 mg kg^−1^ (FERT-200); in the 30–60 cm soil depth, the concentration of soil mineral N varied from 25.20 mg kg^−1^ (CONT) to 64.88 mg kg^−1^ (FERT-200) (Figure 4c).

### 3.5. Acidity and Electrical Conductivity in the Soil

**Figure 5:**
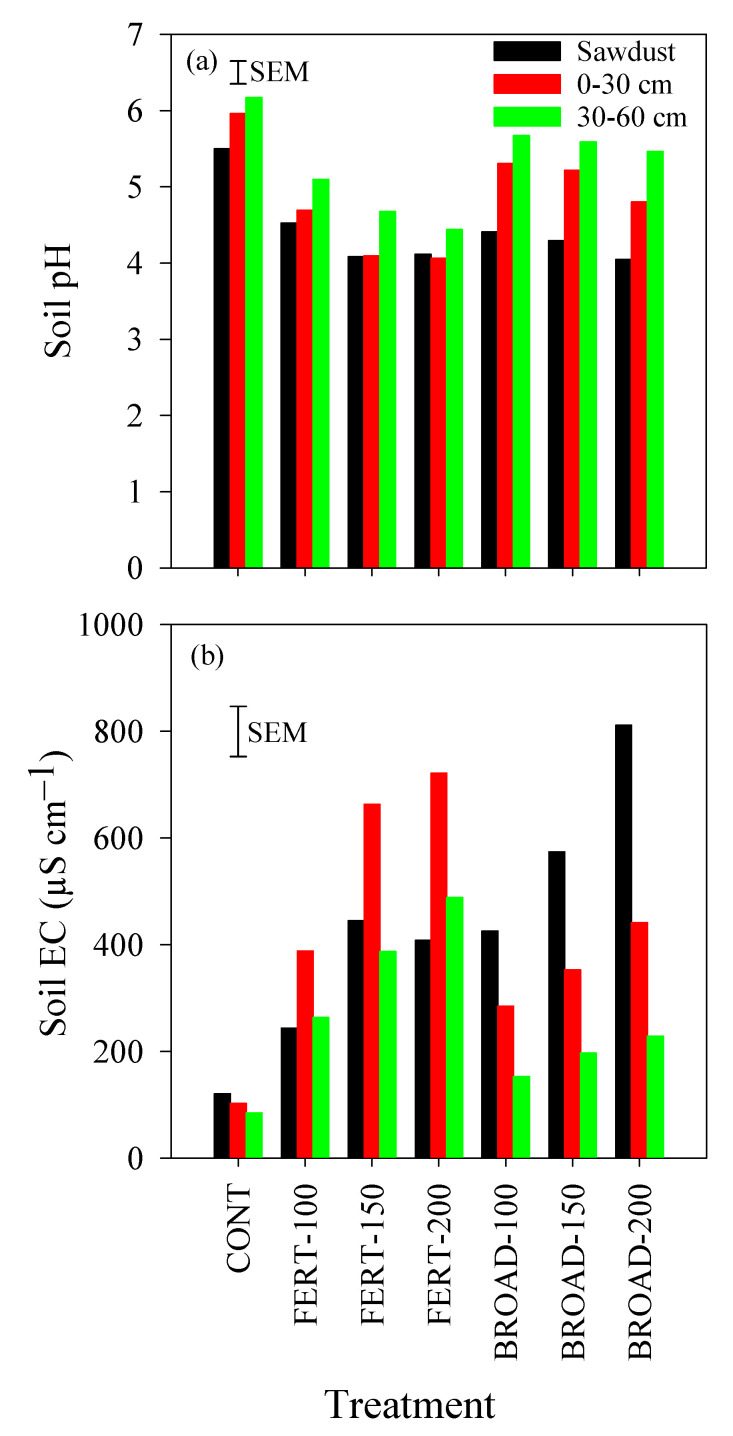
(**a**) Soil pH and (**b**) electrical conductivity (EC) at 0–30 cm and 30–60 cm soil depth and in the sawdust mulch layer with annual applications of N through fertigation (FERT-100, 100%; FERT-150, 150%; FERT-200, 200%) and broadcast (BROAD-100, 100%; BROAD-150, 150%; BROAD-200, 200%) methods to highbush blueberry (*Vaccinium corymbosum*) in fall 2018. Error bars represent the standard errors of the means (SEM) for comparing all values (n = 28 and df = 21).

The soil pH was significantly influenced by N rates, but the extent varied with depth (*p* < 0.001). More specifically, there was a linear trend of soil pH with N application rates (Table 2). In the sawdust layer, soil pH varied from 5.62 (CONT) to 4.00 (FERT-200); In the 0–30 cm depth, soil pH varied from 5.97 (CONT) to 4.04 (FERT-200); and in the 30–60 cm depth, soil pH varied from 6.17 (CONT) to 4.48 (FERT-200) (Figure 5a). Orthogonal contrast comparisons showed that soil pH remained high under CONT compared with all N treatments (Table 2), while FERT-based treatments had lower pH values (soil pH was 4.22, 4.28 and 4.72 in the sawdust, 0–30 cm depth and 30–60 cm depth, respectively) compared with BROAD-based treatments (soil pH was 4.32, 5.10 and 5.58 in the sawdust, 0–30 cm depth and 30–60 cm depth, respectively) (Figure 5a).

Soil EC was significantly influenced by N rates, but the extent varied with depth (*p* < 0.001). More specifically, there was a linear trend of soil EC with N application rates (Table 2). In the sawdust layer, soil EC varied from 125.2 µS cm^−1^ (CONT) to 724.5 µS cm^−1^ (BROAD-200); in the 0–30 cm depth, soil EC varied from 110.8 µS cm^−1^ (CONT) to 789.3 µS cm^−1^ (FERT-200); and in the 30–60 cm depth, soil EC varied from 96.2 µS cm^−1^ (CONT) to 518.3 µS cm^−1^ (FERT-200) (Figure 5b). Orthogonal contrast comparisons showed that soil EC remained low under CONT compared with all N treatments (Table 2), while FERT-based treatments had higher EC values (soil EC was 623.4 and 384.6 in the 0–30 cm depth and 30–60 cm depth, respectively) compared with BROAD-based treatments (soil EC was 361.3 and 193.7 in the 0–30 cm depth and 30–60 cm depth, respectively), except in the sawdust layer (Figure 5b).

### 3.6. Blueberry Yields

**Figure 6:**
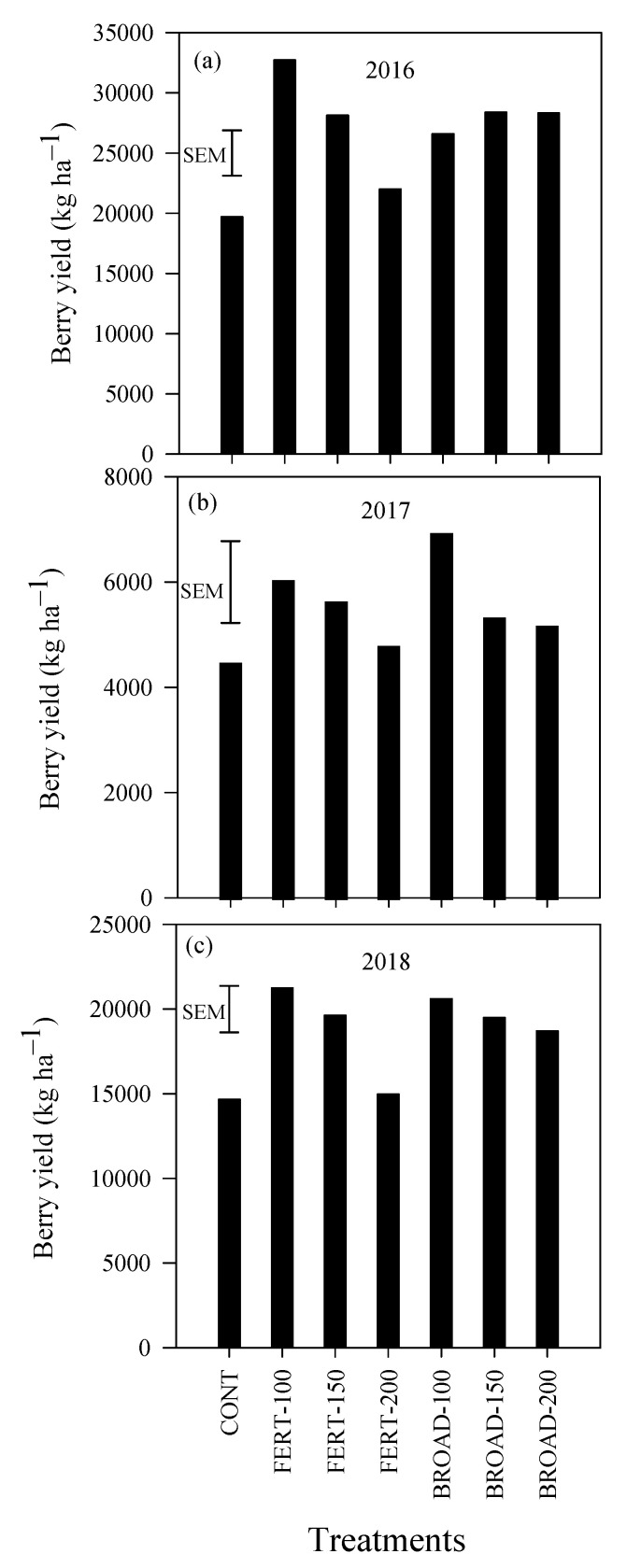
Total berry yield (kg ha^−1^) in (**a**) 2016, (**b**) 2017 and (**c**) 2018 with annual applications of N through fertigation (FERT-100, 100%; FERT-150, 150%; FERT-200, 200%) and broadcast (BROAD-100, 100%; BROAD-150, 150%; BROAD-200, 200%) methods to highbush blueberry (*Vaccinium corymbosum*). Error bars represent the standard errors of the means (SEM) for comparing all values (n = 28 and df = 21).

Berry yield was significantly influenced by N application methods and rates in 2016 (*p* < 0.001), but not in 2017 (*p* = 0.929) or 2018 (*p* = 0.336). In 2016, berry yield was 19,698 kg ha^−1^ under CONT, which was significantly lower compared with N additions, except FERT-200 (Figure 6a). On average, berry yield increased by 66% with FERT-100, 43% with FERT-150 and only 12% with FERT-200 compared with CONT (Figure 6a). In the same year, berry yield increased by 35% with BROAD-100 and 44% with BROAD-150 and BROAD-200 compared with CONT. Berry yield averaged 5278 kg ha^−1^ in 2017 and 18,817 kg ha^−1^ in 2018 (Figure 6b,c). A detailed examination of berry yield among N application methods and rates showed a decreasing trend with increasing N application with FERT- and BROAD-based treatments in 2017 and 2018. For example, berry yield was 5643 kg ha^−1^ with FERT-150 but 4801 kg ha^−1^ with FERT-200, and 6939 with BROAD-100 but 5186 kg ha^−1^ with BROAD-200 (Figure 6b) in 2017. In addition, berry yield was 21,253 kg ha^−1^ with FERT-100, but 14,979 kg ha^−1^ with FERT-200, and 20,607 kg ha^−1^ with BROAD-100, but 18,713 kg ha^−1^ with BROAD-200 (Figure 6c) in 2018.

### 3.7. Relationships between Soil And Leachate Chemical Properties and Berry Yield

**Figure 7:**
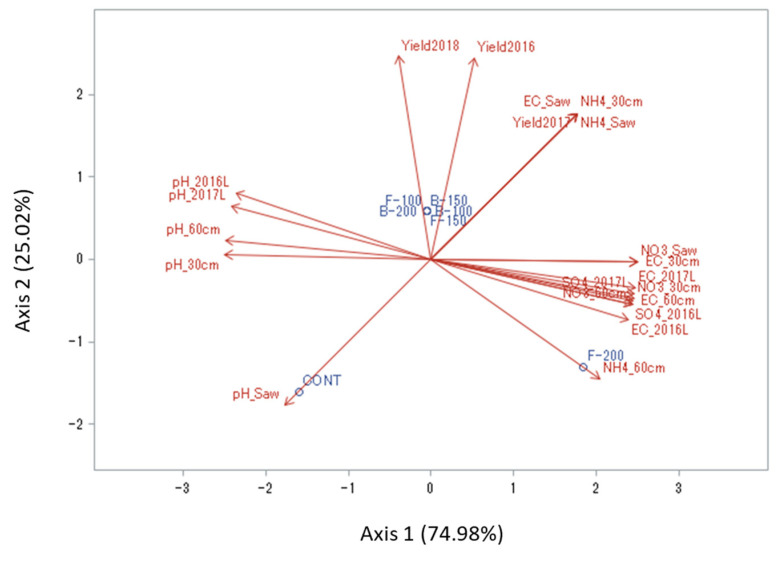
Correlations between berry yield and soil and leachate chemical properties as tested by principal component analysis (PCA) (Yield2016, Yield2017, Yield2018, blueberry yield in 2016, 2017 and 2018, respectively; NO3_Saw, NO3_30 cm, NO3_60 cm, soil nitrate N concentrations in the sawdust layer and 0–30 cm and 30–60 cm depth; NH4_Saw, NH4_30 cm, NH4_60 cm, soil ammonium N concentrations in the sawdust layer and 0–30 cm and 30–60 cm depth; pH_Saw, pH_30 cm pH_60 cm, soil pH in the sawdust layer and 0–30 cm and 30–60 cm depth; EC_Saw, EC_30 cm, EC_60 cm, electrical conductivity in the sawdust layer and 0–30 cm and 30–60 cm depth; EC_2016L, EC_2017 L, electrical conductivity in the leachate collected in the 2016–2017 and 2017–2018 periods; SO4_2016 L, SO4_2017 L, sulfate concentrations in the leachate collected in the 2016–2017 and 2017–2018 periods; pH_2016 L, pH_2017 L, acidity in the leachate collected in the 2016–2017 and 2017–2018 periods).

Principal component analysis (PCA) showed that N applications significantly influenced leachate and soil chemical properties, including NH_4_^+^-N, NO_3_^−^-N, SO_4_^−^-S, pH, EC and berry yield (Figure 7). The N applications were separated into three groups for their effects on chemical properties and yield, including (I) CONT, (II) FERT-100, FERT-150, BROAD-100, BROAD-150 and BROAD-200 and (III) FERT-200. Groups I and III negatively affected berry yields during the three years of study, but not group II. Pearson’s correlation among berry yields and chemical properties of soil and leachate (NH_4_^+^-N, NO_3_^−^-N, SO_4_^−^-S, pH, EC) were in line with the results of PCA (Table S1). The concentration of NH_4_^+^-N in the sawdust layer and 0–30 cm depth were positively correlated with berry yields (Table S1). The concentrations of NO_3_^−^-N and SO_4_^−^-S in the soil and leachates were positively correlated with EC in the soil and leachate, but negatively correlated with pH in the soil and leachate (Table S1).

## 4. Discussion

### 4.1. Nutrient Concentrations in Leachates

High rates of N fertilizer coupled with irrigation are generally associated with high nutrients leaching losses in horticultural crops [21]. Studies showed that N fertilizer applications higher than optimal rates can lead to linear or even exponential increases in the concentrations of NO_3_^−^-N in leachate [20,21]. Our results are consistent with the literature, as the concentrations of NO_3_^−^-N, NH_4_^+^-N and SO_4_^−^-S in the leachates increased with increasing N fertilizer rates during the 2016/17 and 2017/18 production years (Figure 2a–c). High concentrations of nutrients in the leachate were particularly pronounced during the period of 9 May to 19 September. This period coincided with low precipitation and high temperatures (Figure 1), and therefore irrigation was applied to offset the evapotranspiration. Irrigation is widely used in highbush blueberry production because plants are shallow rooted [19,22]. However, irrigation triggered the downward transport of nutrients present in the rhizosphere, causing fertilizer losses and threatening the quality of groundwater. In this study, high concentrations of nutrients in the leachate were obtained under FERT compared with BROAD management although both systems were irrigated in the same way. Under FERT management, dissolved fertilizers are distributed along with irrigation water. Fertigation makes it easier for dissolved fertilizers to move downward with irrigation water through the layer of sawdust mulch covering the raised bed and into the rhizosphere and the deeper soil layer [13]. In contrast, under BROAD management, fertilizer granules are applied on the surface of the sawdust mulch. Sawdust mulch is relatively permeable to water flow [19] and much of the water available for plants originated from irrigation due to limited precipitation in the summer. Only a portion of fertilizer granules was dissolved and transported downward through the layer of sawdust mulch into the rhizosphere. In fact, it is known that high concentrations of NH_4_^+^-N remain in the sawdust layer at the end of the growing season [13]. The persistence of NH_4_^+^-N in the sawdust layer is thus the result of residual fertilizer granules and a low nitrification rate. Interestingly, during the period of November to April, the concentrations of nutrients in the leachates under FERT management decreased, while BROAD management experienced a mini wave of high concentrations of nutrients in the leachates, particularly NO_3_^−^-N and SO_4_^−^-S (Figure 1 and Figure 2a,b,e,f). The residual fertilizer granules retained in the sawdust mulch are dissolved by the heavy precipitation occurring in the fall and winter, thus facilitating the downward transport of nutrients through the layer of sawdust down to deeper layers. This second wave was associated with BROAD-150 and BROAD-200, with concentrations of NO_3_^−^-N and SO_4_^−^-S in the leachates catching up to those of FERT-150 and FERT-200, particularly in the winter of the 2017/18 period (Figure 2a,b,e,f). During the period of October to February, 868 mm of precipitation were obtained in 2016/17 compared with 1104 mm in 2017/18 (Figure 1a,b). This indicates that the extra amount of precipitation obtained during the latter year contributed to higher concentrations of nutrients in the leachates compared with the year before. Several studies showed that NO_3_^−^-N concentrations in the leachates are higher following heavy rainfall and are therefore more closely tied to precipitation patterns [31,32].

In summary, nutrients leaching under FERT management increased with fertilizer rates and irrigation water during the dry period of the growing season and decreased in the off-season. In contrast, BROAD management experienced two periods with high concentrations of nutrients in the leachate: the first in the summer, when the plots were irrigated regularly, and the second over the winter, when the region experienced a large amount of precipitation (Figure 2a,b,e,f). Our results are important as they provide evidence of varying patterns in nutrients leaching in blueberry production systems under FERT and BROAD management. Nutrient leaching under FERT management is high and peaked during the summer owing to irrigation and decreased in the fall and winter despite heavy precipitation. Nutrient leaching under BROAD management is on average lower than FERT management, but occurred in summer owing to irrigation and in fall and winter owing to heavy precipitation. In contrast to our findings, Kowalenko [33] found that in the lower mainland of BC, there is a low risk of NO_3_^−^-N leaching during the spring and summer, but a high risk during fall and winter months due to the substantial rainfall the area receives at this time. Our study presents temporal patterns that need to be accounted for to track nutrient leaching in blueberry production systems in BC.

Considering Canadian regulations on NO_3_^−^-N concentrations in drinking water, the concentrations of NO_3_^−^-N in the leachates were more than 10 times the critical limit of 10 mg L^−1^ [7] for FERT-150 and FERT-200 and between 5 and 10 times for FERT-100 (Figure 2a). During the same period, the concentration of NO_3_^−^-N in the leachates was generally slightly above the critical limit for BROAD-100, twice the critical limit for BROAD-150 and up to four times for BROAD-200. These figures are also above standard concentrations of NO_3_^−^-N in groundwater from other countries and institutions, including the World Health Organization, 50 mg L^−1^ [34], the European Union, 50 mg L^−1^ [35] and US, 45 mg L^−1^ [8]. There are concerns spanning nearly 50 years on the contamination of the transboundary Abbotsford-Sumas Aquifer, which is a major drinking source for both Canadians and Americans [36]. The survey showed that between 2003 and 2013, NO_3_^−^-N concentrations in the Abbotsford-Sumas Aquifer increased from 10.7 mg L^−1^ to 73 mg L^−1^ [36]. Sustainable N fertilization and irrigation are therefore needed to reduce the concentrations of NO_3_^−^-N in the leachates in blueberry production systems in southern BC.

The concentrations of NH_4_^+^-N in the leachates are generally low because the positively charged nutrient is fixed by soil colloids [9]. However, there is a growing concern about ammonia contamination in freshwater since concentrations of total ammonia greater than 1.0 mg L^−1^ can be toxic to aquatic and semiaquatic plant species [10]. Our study showed high concentrations of NH_4_^+^-N in the leachates with FERT-200 during the summer period or 9 May to 19 September (Figure 2c,d). Concentrations of NH_4_^+^-N in the leachates as high as 4 mg L^−1^ with FERT-100 and 8 mg L^−1^ with FERT-150 were also measured, although during a limited period in the summer (20 June to 31 July). High concentrations of NH_4_^+^-N with FERT management indicate that some portion of NH_4_^+^-N dissolved in the FERT water was not taken up by plants or fixed by soil colloids. This can be explained by NH_4_^+^’s ability to quickly move through the sawdust layer under FERT when irrigation is applied [22]. BROAD-based applications release NH_4_^+^ from fertilizer granules that move more slowly through the sawdust mulch, in which NH_4_^+^ is more susceptible to immobilization and less likely to be leached [22].

Guidelines for Canadian drinking water quality suggest that SO_4_^2−^-S concentrations below 500 mg L^−1^ are safe for consumption [7]. This critical limit was not reached in our study, where maximum concentrations of SO_4_^2−^-S in the leachates were between 250 and 350 mg L^−1^ in both years (Figure 2e,f). On the other hand, increases in SO_4_^2−^-S concentrations as small as 2 mmol L^−1^, (192.12 mg L^−1^) have been found to negatively impact freshwater ecosystems [10]. Sulfate can enter freshwater systems by leaching into the surface or groundwater and has the potential to mobilize phosphorus, resulting in increased phosphate concentrations, which lead to eutrophication [37]. In addition, high levels of SO_4_^2−^-S pollution in freshwater systems can cause increases in the anaerobic decomposition of organic matter or result in sulfide toxicity when reduced [37]. Adjusting N fertilizer applications to optimal rates and the use of different sources of fertilizer N such as urea in blueberry production systems will further decrease the concentrations of SO_4_^2−^-S in leachates and subsequently in groundwater and freshwater systems.

### 4.2. Yield

The present study highlights decreased berry yield with N applications above recommended rates under BROAD management. This pattern is evidenced during the 10th and 11th year of production (Figure 6b,c). The decreased berry yield under fertigation with N applications above recommended rates was also observed in previous production periods [13] and was therefore carried through the 9th and 11th production years. Reasons for berry yield decrease with N applications above recommended rates include high EC and acidity [13]. These changes in chemical soil properties occurred in this long-term blueberry experiment because of the use of ammonium sulphate as a source of N. The use of the large amount of N fertilizers above recommended rates has the potential to limit berry production and the lifespan of blueberry plantings regardless of the method of N applications. Reduced berry yield obtained in the 10th production year is due to the heavy pruning performed during the fall of 2016. This practice is commonly used by blueberry farmers to rejuvenate the shrubs and prolong their life span [26]. The significant trends of berry yield decrease above recommended rates that was obtained under FERT-based treatments in previous years [13] may have been altered by the heavy pruning of fall 2016.

### 4.3. Relationship between Yield and the Nutrient Concentration in Leachate and Soil

The results of PCA showed that berry yields were positively correlated with concentrations of NH_4_^+^-N in the sawdust layer and 0–30 cm depth (Figure 7). It is well known that blueberry plants prefer NH_4_^+^-N over NO_3_^−^-N [17] and therefore high concentrations of NH_4_^+^-N in the sawdust layer and 0–30 cm depth are favorable for blueberry N uptake, growth and productivity. Berry yields were also negatively correlated with the pH of the sawdust mulch, particularly under CONT, where persistent high pH values are not suitable to blueberry growth. This suggests the need for elemental sulfur in these plots to decrease the pH between 4.2 and 5.5, which is recommended for blueberry plantings [17]. A negative correlation was obtained between berry yields and the concentrations of NH_4_^+^-N in the 30–60 cm soil depth, particularly under FERT-200, probably due to the extensive leaching of this N form which induced low concentrations, as well as with sawdust pH. Negative correlations were also observed between yield, soil and leachate pH. On the other hand, pH was negatively related to soil EC, NO_3_^−^-N and SO_4_^2−^-S. These correlations highlight how closely acidity is related to the nitrification process. In addition, the dissolution of ammonium sulfate fertilizer releases SO_4_^2−^ which accumulates in the soil and leachate, causing increased EC values [38,39]. In our study, EC values above critical limits were only obtained under FERT-200. These observations are in line with previous studies including those published from this long-term site [13,18]. Bryla and Machado [40] observed that large amounts of ammonium sulfate fertilizers result in increased soil EC and salt stress in blueberry plants, and consequently yield reduction.

## 5. Conclusions

Nutrients (NO_3_^−^-N, NH_4_^+^-N and SO_4_^−^-S) leaching in this long-term blueberry field was increased by N applications during the 2016/17 and 2017/18 production years. High concentrations of nutrients in the leachate were pronounced during the summer due to irrigation. FERT and BROAD management increased the concentrations of NO_3_^−^-N in the leachates between 2 and 10 times the standard recommendations for groundwater quality. BROAD management highlighted two waves of high NO_3_^−^-N in the leachates, the first in the summer due to irrigation water and the second in the fall and winter associated with heavy precipitations. Our results also indicate a yield decrease with N applications above recommended rates under BROAD management as previously observed under FERT management. Accumulation of mineral N in the soil and its ripple effect on pH and EC negatively affect berry yield with time. It is therefore important to tailor N management to plant needs with broadcast or fertigation application to limit nutrients leaching and to sustain the long-term productivity of blueberry farms.

## Figures and Tables

**Table 1:** Results of ANOVA for chemical properties of leachate water collected in lysimeters (76 cm depth) in the long-term blueberry experiment during the period March 2016 and April 2018 (n = 420 and 555 for March 2016–February 2017 and March 2017–April 2018, respectively).

	March 2016–February 2017					March 2017–April 2018				
	NO_3_^−^-N	NH_4_^+^-N	SO_4_^−^-S	pH	EC ^1^	NO_3_^−^-N	NH_4_^+^-N	SO_4_^−^-S	pH	EC ^1^
	(mg L^−1^)	(mg L^−1^)	(mg L^−1^)		(µS cm^−1^)	(mg L^−1^)	(mg L^−1^)	(mg L^−1^)		(µS cm^−1^)
Sources of variation	*p* values ^2^									
Treatment (T)	<0.001	<0.001	<0.001	<0.001	<0.001	<0.001	<0.001	<0.001	<0.001	<0.001
Sampling periods (SP)	<0.001	<0.001	<0.001	<0.001	<0.001	<0.001	<0.001	<0.001	<0.001	<0.001
T × SP	<0.001	<0.001	<0.001	<0.001	<0.001	<0.001	<0.001	<0.001	<0.001	<0.001
Contrast										
CONT vs. All	<0.001	0.146	0.001	<0.001	<0.001	<0.001	0.034	<0.001	0.002	<0.001
FERT vs. BROAD	<0.001	0.001	<0.001	<0.001	<0.001	<0.001	<0.001	<0.001	<0.001	<0.001
Linear	0.001	0.852	0.032	0.001	0.003	0.004	0.694	0.003	0.004	<0.001
Quadratic	<0.001	0.019	0.004	<0.001	<0.001	0.008	0.002	0.002	<0.002	0.006

^1^ Electrical conductivity; ^2^ probability values.

**Table 2:** Results of ANOVA for the effects of nitrogen applications through fertigation (FERT) and broadcast (BROAD) methods and soil depth (sawdust layer, 0–30 cm and 30–60 cm) on the concentrations of ammonium N (NH_4_^+^-N), nitrate N (NO_3_^−^-N), mineral N (NH_4_^+^-N + NO_3_^−^-N), pH and electrical conductivity (EC) in fall 2018 in a highbush blueberry (*Vaccinium corymbosum*) (n = 84).

	NH_4_^+^-N (mg kg^−1^)	NO_3_^−^-N (mg kg^−1^)	Mineral N (NH_4_^+^-N + NO_3_^−^-N, mg kg^−1^)	pH	EC
Sources of variation	*p* values ^1^				
N applications (N)	0.039	<0.001	0.016	<0.001	<0.001
Depth	<0.001	<0.001	<0.001	<0.001	<0.001
N applications × Depth	0.002	0.020	0.002	<0.001	<0.001
Contrast					
CONT ^2^ vs. All	0.882	0.005	0.114	0.003	0.045
FERT vs. BROAD	0.029	<0.001	0.881	<0.001	0.007
Linear	0.477	0.045	0.015	<0.001	0.001
Quadratic	0.676	0.025	0.097	0.002	0.027

^1^ Probability values; ^2^ control, 0 kg N ha^−1^.

## Supplementary Materials

The following are available online at https://www.mdpi.com/2223-7747/9/11/1530/s1, Table S1: Pearson’s correlation matrix among berry yields and chemical properties of soil and leachate.
