# Supplementary material for: Nutrients Leaching in Response to Long-Term Fertigation and Broadcast Nitrogen in Blueberry Production
Source: Plants (Basel). 2020 Nov 10;9(11):1530. doi: 10.3390/plants9111530 (PMC7696231; doi:10.3390/plants9111530)
Supplement: Supplementary file 1 [file plants-09-01530-s001.pdf]

Table S1. Pearson's correlation matrix **among berry yields and chemical properties of soil and leachate.**

	Yield2016	Yield2017	Yield2018	NO3_Saw	NO3_30cm	NO3_60cm	NH4_Saw	NH4_30cm	NH4_60cm	pH_Saw	pH_30cm	pH_60cm	EC_Saw	EC_30cm	EC_60cm	EC_2016L	EC_2017L	SO4_2016L	SO4_2017L	pH_2016L	pH_2017L
**Yield2016**	1.000																				
**Yield2017**	0.168	1.000																			
**Yield2018**	0.828	0.435	1.000																		
**NO3_Saw**	0.061	-0.067	-0.392	1.000																	
**NO3_30cm**	-0.007	-0.159	-0.439	0.978	1.000																
**NO3_60cm**	-0.238	-0.198	-0.641	0.931	0.927	1.000															
**NH4_Saw**	0.324	0.229	0.171	0.000	-0.173	-0.145	1.000														
**NH4_30cm**	0.101	0.084	-0.309	0.713	0.613	0.571	0.634	1.000													
**NH4_60cm**	-0.132	-0.295	-0.473	0.430	0.487	0.475	-0.154	0.326	1.000												
**pH_Saw**	-0.432	-0.361	-0.054	**-0.806**	**-0.718**	-0.5816	-0.369	**-0.809**	-0.329	1.000											
**pH_30cm**	-0.302	0.028	0.142	**-0.939**	**-0.943**	**-0.7811**	0.068	**-0.627**	-0.457	0.848	1.000										
**pH_60cm**	-0.258	0.074	0.167	**-0.939**	**-0.957**	**-0.8061**	0.170	**-0.549**	-0.415	0.785	0.991	1.000									
**EC_Saw**	0.319	0.361	0.045	0.390	0.243	0.1873	**0.858**	**0.883**	0.163	-0.749	-0.362	-0.249	1.000								
**EC_30cm**	0.117	-0.080	-0.335	**0.988**	**0.984**	**0.8828**	-0.025	**0.709**	0.438	-0.817	**-0.971**	**-0.967**	0.387	1.000							
**EC_60cm**	0.002	-0.223	-0.456	**0.979**	**0.994**	**0.9445**	-0.156	**0.606**	0.501	-0.696	**-0.930**	**-0.946**	0.231	0.975	1.000						
**EC_2016L**	-0.053	-0.218	-0.463	**0.954**	**0.987**	**0.9423**	-0.280	0.504	0.453	-0.627	**-0.906**	**-0.938**	0.108	**0.950**	**0.989**	1.000					
**EC_2017L**	0.120	-0.140	-0.308	**0.962**	**0.985**	**0.8609**	-0.159	0.600	0.404	-0.744	**-0.970**	**-0.983**	0.251	**0.986**	**0.974**	**0.968**	1.000				
**SO4_2016L**	-0.062	-0.192	-0.504	**0.980**	**0.988**	**0.9700**	-0.151	0.609	0.486	-0.684	**-0.903**	**-0.922**	0.231	**0.964**	**0.995**	**0.987**	**0.957**	1.000			
**SO4_2017L**	0.023	-0.129	-0.396	**0.980**	**0.996**	**0.9183**	-0.163	0.607	0.411	-0.727	**-0.946**	**-0.963**	0.242	**0.986**	**0.989**	**0.986**	**0.991**	0.983	1.000		
**pH_2016L**	0.054	0.175	0.419	**-0.926**	**-0.977**	**-0.8991**	0.359	-0.445	-0.415	0.605	**0.904**	**0.943**	-0.050	**-0.936**	**-0.967**	**-0.990**	**-0.969**	-0.961	-0.979	1.000	
**pH_2017L**	-0.108	0.185	0.278	**-0.917**	**-0.962**	**-0.8480**	0.333	-0.425	-0.369	0.637	**0.936**	**0.973**	-0.058	**-0.937**	**-0.956**	**-0.974**	**-0.977**	-0.938	-0.970	0.985	1.000

Yield2016, Yield2017, Yield2018, Blueberry yield in 2016, 2017 and 2018 respectively;
NO3_Saw, NO3_30cm, NO3_60cm, soil Nitrate N concentrations in the sawdust layer and 0-30cm and 30-60cm depth;
NH4_Saw, NH4_30cm, NH4_60cm, soil ammonium N concentrations in the sawdust layer and 0-30cm and 30-60cm depth;
pH_Saw, pH_30cm pH_60cm, soil pH in the sawdust layer and 0-30cm and 30-60cm depth;
EC_Saw, EC_30cm, EC_60cm, Electrical conductivity in the sawdust layer and 0-30cm and 30-60cm depth;
EC_2016L, EC_2017L, Electrical conductivity in the leachate collected in the 2016-2017 and 2017-2018 periods;
SO4_2016L, SO4_2017L, Sulfate concentrations in the leachate collected in the 2016-2017 and 2017-2018 periods;
pH_2016L, pH_2017L, Acidity in the leachate collected in the 2016-2017 and 2017-2018 periods;
